# “I can lose sight of my own well-being because I’m just so focused on them”: a qualitative investigation of eating disorder clinicians’ experiences in England

**DOI:** 10.1186/s40337-026-01615-9

**Published:** 2026-05-02

**Authors:** Kat Novogrudsky, Caroline Le Luel, Janet Treasure, Ulrike Schmidt

**Affiliations:** 1 Centre for Research in Eating and Weight Disorders, King’s College London, London, UK; 2 South London and Maudsley NHS Foundation Trust, London, UK

**Keywords:** Eating disorders, Burnout, Occupational health, Mental health services, Moral injury

## Abstract

**Background:**

Work in eating disorder (ED) services presents unique challenges and rewards that may affect clinicians’ work-related and personal wellbeing. However, research on ED clinician needs, views, and experiences is still sparse, despite major service changes since the COVID pandemic. This study aims to explore and conceptualise NHS ED clinicians’ work-related experiences, challenges, and needs, in order to inform future clinicians wellbeing and service improvement strategies.

**Methods:**

Clinicians working in ED services (*N* = 19) were interviewed using a semi-structured interview guide that probed their professional experiences, work-related needs, and views. Interviews were analysed using NVivo, following guidance from Braun and Clarke (2006) for reflexive thematic analysis.

**Results:**

A holistic ecological systems framework for ED services was created, comprised of five levels of influence: intrinsic, intra-personal, departmental, systemic, and societal. These levels contain nine themes: [1] clinician motivation for working in ED services [2], complexities of ED management [3], clinician personality and emotional disposition [4], team dynamics [5], supervision, management, and organizational support [6], service-level concerns [7], macro-level systemic concerns [8], broader societal challenges in ED care, and [9] COVID-related challenges. Key concerns included the chronic nature and risk of EDs, growing service demands amid limited resources, and regulation through guidelines and commissioning targets.

**Conclusions:**

This presented framework illustrates the multifaceted array of complexities faced by ED clinicians. The interplay of personal, inter-personal, and systemic factors is explored, with clinicians’ interest in and commitment to ED care at the core of the framework. These areas can be targeted to improve clinician job satisfaction and reduce burnout risk, with the goal to provide optimal patient care.

**Supplementary Information:**

The online version contains supplementary material available at 10.1186/s40337-026-01615-9.

## Abstract (plain language summary)

This study explores the experiences and wellbeing of clinicians working in NHS eating disorder (ED) services. Through interviews with clinicians, the research explored both the positive and difficult parts of their job. While staff felt strongly committed to helping people with EDs, many also described feeling emotionally drained and frustrated. This was often due to high workloads, not enough resources, and long waiting lists. Clinicians found it especially hard when they had to follow strict service rules that didn’t work well for individual patients, and when they had to manage complex medical risks. Supportive teams and good supervision helped some staff cope. Wider problems like staff shortages, poor communication between services, and lack of funding compounded emotional strain. The findings show that ED clinicians urgently need more support, including better resources, more flexible ways of working, and proper training, to give safe, effective care without burning out.

**Supplementary Information:**

The online version contains supplementary material available at 10.1186/s40337-026-01615-9.

## Background

 Clinicians working in eating disorder (ED) services face distinct rewards and challenges that can significantly impact their personal and professional wellbeing [1]. While research in this area has grown, ongoing investigation remains essential given mounting pressures on publicly funded healthcare workforces - such as the UK National Health Service (NHS) - where deeper insight into work-related outcomes can inform strategies to enhance wellbeing, improve patient care, and sustain services.

EDs are common and complex mental illnesses characterised by disturbances in eating behaviours, with heterogeneous presentations, ambivalence about treatment, and high risk of relapse [2]. Globally, the prevalence and burden of EDs has been rising in recent decades [3], with a surge in cases during the COVID-19 pandemic [4]. This surge compounded existing pressures on NHS ED services - including staff absences, rising referrals, reduced capacity, and longer wait times that remain elevated today - placing considerable strain on clinicians and threatening patient care. Understanding ED clinicians’ experiences is therefore vital to identifying current challenges and informing strategies to better support them [5–8].

Alongside systemic pressures, ED clinicians face challenges inherent to the work: illness severity, patient complexity, difficult treatment decisions, high relapse rates, clinical uncertainty, limited resources, and poor coordination of care [1, 9–12]. Although ED clinicians often work in multidisciplinary teams (MDTs), which have been shown to support better outcomes [13], resource constraints and limited support remain persistent challenges [14]. The interplay of these factors - spanning individual clinician characteristics, team dynamics, organisational structures, and broader healthcare system pressures - reflects an ecological understanding of wellbeing. Bronfenbrenner’s ecological systems theory [14], which conceptualises individual functioning as shaped by nested layers of environment, provides a useful framework for understanding how these multilevel influences interact to affect ED clinicians’ experiences.

These challenges are associated with a range of adverse work-related outcomes. Burnout -characterised by emotional exhaustion, depersonalisation, and reduced personal accomplishment [15] - is a significant concern in ED settings, linked to longer hours, insufficient resources, high clinical demands, and the inherent chronicity and relapse burden of EDs [1, 9]. Burnout has downstream consequences for both clinician health and patient care, including impaired decision-making and changes in therapeutic engagement [16, 17]. Relatedly, problematic or negative countertransference - the clinician’s emotional responses toward patients, shaped by their own experiences and vulnerabilities [19] - may contribute to emotional exhaustion through reactions such as frustration, overinvestment, or hopelessness [18–20]. Clinicians may also experience moral injury - the psychological distress arising from situations that conflict with one’s professional values [21, 22], which has been documented in around one quarter of healthcare professionals and is associated with negative self-appraisal and mental health difficulties [26]. During the pandemic, NHS clinicians reported high rates of mental health difficulties, with 10% reporting suicidal ideation, which was linked to morally injurious events, lack of managerial support, and compromised care [27] — underscoring the seriousness of these pressures for ED clinicians specifically.

Clinician wellbeing and the effective administration of services are integral to patient care. When clinicians experience high levels of stress, burnout, or poor support, this can negatively impact therapeutic engagement, decision-making, and the quality of care delivered [23]. Conversely, supporting clinician wellbeing enhances staff retention, fosters sustainable service delivery, and ultimately benefits patients. Focusing on clinicians’ experiences therefore not only addresses the needs of staff but also contributes to improving service provision and patient outcomes [23].

Research on clinician wellbeing in ED settings remains limited; a few recent studies exist [15, 21], but most were conducted before the pandemic. A recent systematic scoping review by Novogrudsky et al. (2025) provided a useful synthesis of the field, although research specifically examining ED clinicians’ unique working experiences post-pandemic remains limited. Furthermore, as services have faced drastic changes during and following the pandemic – including widespread adoption of remote consultations, reconfiguration of patient care pathways, and acute staffing shortages [6, 7] - it is important to understand how clinical needs have also been affected and what may need to be addressed presently. More broadly, there needs to be insight into the needs of clinicians working in the public healthcare system.

Using Bronfenbrenner’s ecological systems theory as an organizing framework, this qualitative study investigates the wellbeing, needs, and experiences of NHS England ED clinicians. It examines how individual clinician perspectives interact with broader contextual challenges (including the lasting impacts of the COVID-19 pandemic) to identify current workplace difficulties and determine how to better support these frontline teams and services. Wellbeing is used as an umbrella term encompassing mental, social, occupational and physical functioning.

### Research questions

How do ED clinicians perceive their roles, and how do these perceptions impact their wellbeing?

What are ED clinicians’ experiences of working within their teams, and what support do they feel is needed?

How have services and clinicians’ experiences changed since the Covid-19 pandemic, and what challenges remain?

## Methods

### Ethics

This study was granted ethical approval by the KCL Health Faculties (Blue) Research Ethics Subcommittee, HR/DP-22/23-30046. Considering the potentially distressing content of some questions, participants were provided with a range of outside support sources as a precautionary measure, in case further support was needed.

### Participants and recruitment

This study used a convenience sampling approach, inviting the attendees of a national training course for adult community NHS ED services in England [24]. Course attendees (N = ~ 600) were sent an invitation email to which they could respond if they were interested in completing an interview on their work-related experiences. Some clinicians also forwarded the invitation to their colleagues; hence the total number of email recipients is unknown. Nineteen healthcare professionals working in various NHS ED community teams across England were recruited. Table 1 presents participant characteristics, including role type, service setting, and years of experience. Notably, most participants had 1–3 years of experience in their current role, and most participants were Female (*N* = 17).

Sample adequacy was guided by the concept of information power (Malterud et al., 2016) which posits that the more relevant information each participant holds in relation to the study aim, the fewer participants are required. This study explores the experiences, needs, and wellbeing of ED clinicians in NHS England through a qualitative lens, examining current work-related challenges and how services and teams might be better supported. Drawing on the interpretive nature of clinicians’ experiences and the contextual challenges within NHS services, shaped in part by the COVID-19 pandemic, it examines current work-related difficulties and how services and teams might be better supported. Wellbeing is used as an umbrella term encompassing mental, social, occupational and physical functioning.

### Interview schedule

A semi-structured interview schedule was created by the authors (see Appendix A). Initial questions focused on clinicians’ careers and professional experiences, followed by questions about professional needs, job satisfaction, and enjoyment. Other questions explored clinicians’ training experiences and needs. If the clinician identified as having a personal or lived experience of having or caring for someone with an ED, questions were asked regarding how their personal experiences shaped their professional lives. The schedule was informed by existing literature on ED clinician wellbeing and developed collaboratively among the research team, which includes experienced ED clinicians.

### Procedure

Clinicians were sent the information sheet (which explained intentions to publish the research in a thesis and in academic journals) and a consent form which was to be returned prior to the agreed interview date. The interviewer (KN) reviewed participants’ questions, reiterated their rights, and provided a brief self-introduction. The semi-structured interview guide was followed, focusing on areas which were most relevant or emotive for the participants. Interviews were recorded, ranging from 24 to 96 min (M = 40; Mdn = 34). Afterward, participants were offered a £20 bank transfer for their participation. Following Tong et al. (2007), the consolidated criteria for reporting qualitative studies (COREQ) checklist is presented in Appendix B [25]. This tool provides a framework to ensure explicit and comprehensive reporting of the interview process.

### Data analysis

Interview transcripts were downloaded from Microsoft Teams. All identifiable information was removed, then the interviews were listened to again and re-transcribed to ensure accuracy. Transcripts were imported into NVivo 14 where they were analysed. Memoing was used throughout coding and theme development to capture insights and reflexive observations. Using collaborative reflexivity, themes, subthemes and labels were discussed and co-created between all authors. Lastly, a summary of the methodology and results were shared with participants. This was intended as an ethical and relational gesture to honour participants’ time and contribution, not as a means of validating the analytic findings, which reflect the researchers’ interpretations of the data.

### Thematic approach

This study employed Reflexive Thematic Analysis (RTA) as outlined by Braun and Clarke (2006), using an inductive and interpretive approach to identify patterns of meaning across the dataset [26]. The analysis was conducted from a critical realist perspective, which assumes that participants’ accounts reflect both their lived experiences and the broader social and organisational contexts in which these are situated. This method was chosen due to its flexibility and acknowledgement of the active role of the researcher in knowledge production. Theme development was primarily inductive and data-driven, while also being informed by theoretical sensitivity to existing concepts relevant to clinician wellbeing and service dynamics. Themes were constructed through a repetitive process of familiarisation, coding, and theme development, with attention to the emotional tone and contexts of participants’ narratives. Following initial theme construction, it became evident that the emerging patterns began to mirror Bronfenbrenner’s Ecological Systems Theory (1979), which was subsequently applied as an interpretive lens rather than a coding framework [14]. This was used to structure and interpret the resulting themes, allowing for a layered understanding of how individual, relational, institutional, and societal factors shaped clinicians’ experiences and motivations.

### Reflexivity

Reflexive thematic analysis requires researchers to critically reflect on their positionality and how their experiences, perspectives, and values inevitably shape and inform the analytic process. KN, who conducted the interviews and data analysis, is a White female student with lived experience of ED and its treatment within the NHS. Her personal experience and aspiration to work as an ED clinician are recognised as having actively informed her interpretations. To ensure interviews were driven by participants’ experiences, KN withheld disclosure of her own lived experience, keeping participants’ accounts central. Each co-author’s positionality is recognised as shaping their analytic contributions: CLL, as a carer of two individuals recovered from anorexia nervosa, brought experiential understanding to data engagement; JT and US, as senior NHS ED clinical academics with experience across inpatient and outpatient settings respectively, contributed clinical depth to the interview guide, sense-checking, and meaning-making. Reflexivity was actively practised through regular supervision meetings and dialogue with co-authors, encouraging critical reflection on interpretive choices and meaning-making. These discussions helped to balance emotional proximity with analytic distance.

## Results

**Fig. 1:**
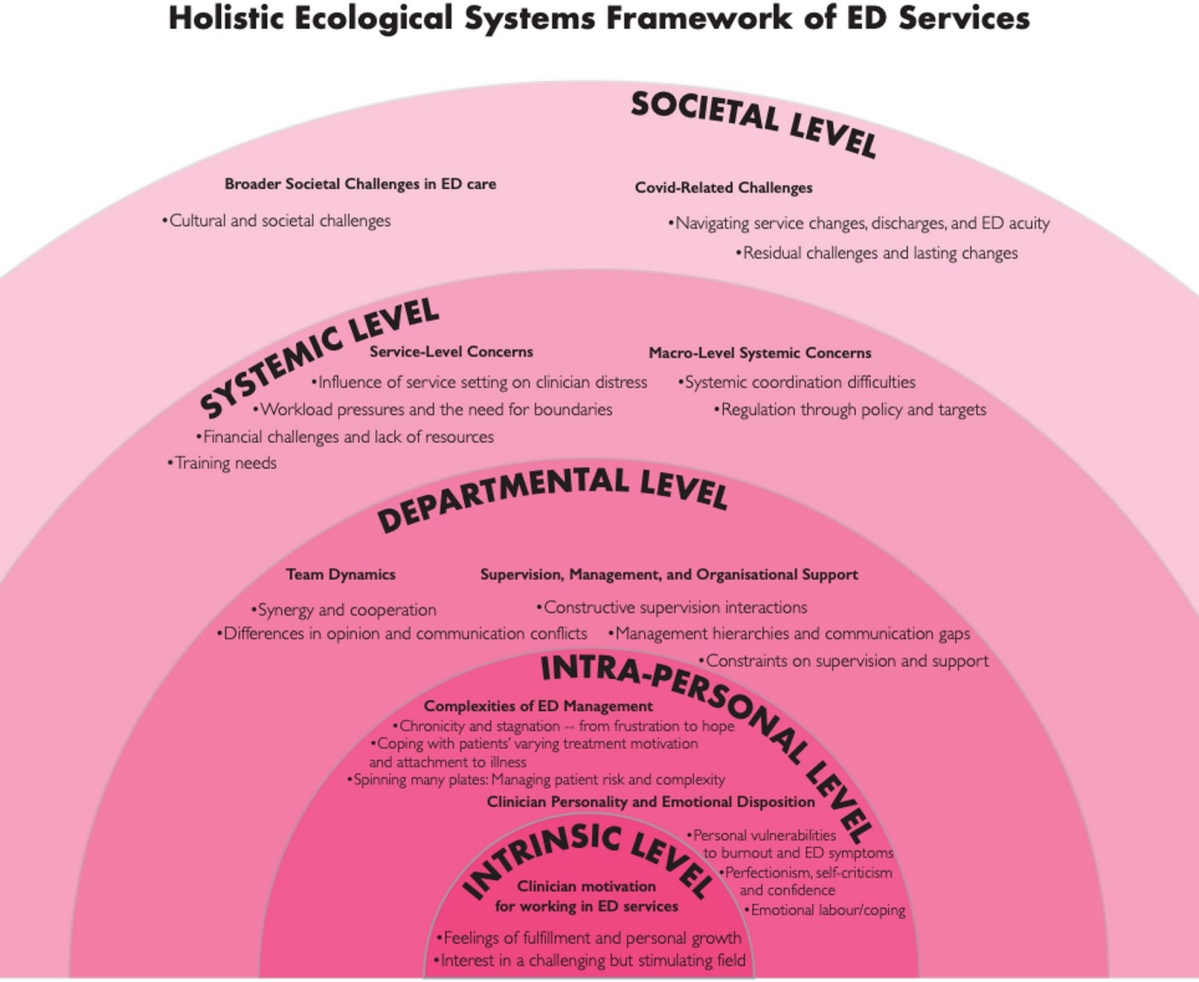
Holistic ecological systems framework of ED services

Nine themes evolved from the data: [1] clinician motivation for working in ED services [2], complexities of ED management [3], clinician personality and emotional disposition [4], team dynamics [5], supervision, management, and organizational support [6], service-level concerns [7], macro-level systemic concerns [8], broader societal challenges in ED care, and [9] COVID-related challenges. These themes were organized into five supra-themes, influenced by Bronfenbrenner’s (1979) Ecological Systems Theory. The themes were shaped into a holistic ecological systems framework to contextualize the current experiences of ED clinicians (see Fig. 1). A coding tree can be found in Supplement 1.

### Theme 1: clinician motivation for working in ED services

#### Subtheme: interest in a challenging yet stimulating and resonant field

Clinicians expressed genuine interest in the field and fondness for patients. Introduced to EDs during training or through personal connections, clinicians felt compelled to learn more and contribute to the field. Many were drawn to the physical–mental health overlap, finding value in addressing both psychological and physical factors in tandem.

*C4: the variety of work that we do*,* it straddles both physical presentations and psychological presentations… The complexity of working with people just provides a richness of work.*

Another aspect that appealed to clinicians was the relatability of EDs. Most (17/19) clinicians in this study were female, and many of their patients were also female. Female clinicians resonated with patients’ struggles, understanding the social pressures on women and how these lead to extreme behaviours to manage societal expectations around appearance for whom this is related to their ED. This fuelled their interest in the field and contributed to feelings of empathy for, and connectedness with their patients.

*C5: It’s also quite relatable*,* a lot of the things that the patients struggle with - just as being a young woman in my 20s. Just seeing how extremely certain things can impact them and the extreme that body concerns can take…*.

#### Subtheme: feelings of fulfilment, learning and personal growth

Clinicians described their work as emotionally demanding yet rewarding. The challenges inherent in the role were integral to the sense of purpose and fulfilment they derived from their work. The opportunity to witness patients’ gradual psychological and behavioural shifts was rewarding. Beyond clinical outcomes, clinicians emphasized the relational element of their work as central to their job satisfaction. The development of a therapeutic alliance was seen as a transformative encounter for both the patient and clinician.

*C4: I help people*,* I facilitate people’s human growth. Getting to learn about people from different walks of life. Truly try to learn about people*,* to share experiences …*.

Clinicians recognised that their fulfilment was intertwined with a sense of moral purpose, reinforcing their commitment to the field despite its emotional weight. Such experiences appeared to sustain clinicians’ sense of meaning and professional identity, serving as a counterbalance to the emotional exhaustion and moral strain.

### Theme 2: complexities of ED management

#### Subtheme: chronicity, stagnation, and treatment ambivalence - from frustration to hope

Clinicians expressed the challenge of combatting the chronicity of EDs, describing the difficult management (especially of longstanding EDs year after year), and the tension of knowing when to persist and when to step back. They often oscillated between empathy and frustration in the face of patient ambivalence, struggling to remain hopeful.

*C6: People who are stuck… that can be challenging because you have to be empathetic to the frustration that people are feeling*,* but it’s not helpful if you end up setting the frustration with them.*

Clinicians described their understanding of EDs as serving an important coping function for patients, which helped explain patients’ reluctance to ‘give up’ behaviours, though highlighting the challenges this created for treatment engagement.

*C19: Sometimes I feel like the bad guy*,* getting people to drop something that’s actually really helped them in some of the most difficult times.*

Clinicians also highlighted some patients’ deception strategies which sometimes evoked feelings of frustration, upsetting the trust between the clinicians and patient.

*C5: I really struggle when people consistently lie about small things that aren’t even to do with food*,* trying to remember that it’s all part of the disorder… one of the biggest challenges is holding on to that and not feeling like people are just lying to you because they don’t trust you or they don’t want you to help them.*

Ambivalence was seen to spread, particularly on inpatient wards, where group environments could either support recovery or reinforce negative behaviours. Clinicians also felt distress when delivering treatment to patients resistant to change, feeling coercive or forceful. Despite these challenges, clinicians remained optimistic, acknowledging that the ED, not the patients, were difficult to treat. Holding hope, keeping focused on recovery helped to reinforce the rewards of the role.

*C9: I think 90% of [treatment] is hard work and draining… sometimes I’ve come out of sessions and I’ve got really upset… it’s just that 10% at the end …You get a lot of satisfaction. That 90% was hard*,* but it was all completely worth it because now you look at this person that is absolutely thriving…*.

### Subtheme: spinning many plates: managing patient risk and complexity

Clinicians felt a persistent tension between their desire to provide compassionate care and the realities of working within a system marked by risk, resource limitations, and structural challenges. One of the most emotionally taxing aspects of their role was the responsibility of managing medical risk, particularly the fear of a patient deteriorating or dying due to treatment gaps. This fear was compounded by concerns over potential media scrutiny and backlash. Such risk contributed to a broader reluctance among other healthcare professionals to engage in ED work, creating collaboration barriers and service gaps. Clinicians often found themselves carrying the weight of systemic shortcomings, navigating complex cases with limited support and heightened emotional strain.

C4: even GPs are sh*t scared. That’s manifested now with medical monitoring pressures and things with all that. At the slightest opportunity where GPs can go ‘Right,* that’s not ours*,* this isn’t general medicine*,* this is ED…’ Yes*,* it’s a lot to do with money*,* but as well… is the fear of ED patients*,* because the perception is that they die.*

Beyond medical risk, clinicians spoke of the multifaceted nature of their role, describing the need to wear ‘many different hats’ as they balanced ED-specific interventions with an awareness of patients’ broader life contexts. The therapeutic space was complicated by patients’ lived realities, including financial hardship and relational instability.

*C19: With outpatient [treatment]*,* sometimes their life is very chaotic… just because they feel motivated*,* there can be so many things in their lives that get in the way.*

Clinicians carried the emotional labour of patients’ disappointment, especially when treatment fell short after long waits. The therapeutic relationship became a site of ethical complexity, where clinicians had to make difficult decisions that prioritized long-term wellbeing over immediate engagement, often at personal emotional cost.

### Theme 3: clinician personality and emotional disposition

#### Subtheme: personal vulnerabilities to burnout and ED symptoms

Many clinicians resonated with their patients’ challenges. While this was motivating for some, it also introduced risks when paired with personal vulnerabilities. For example, some clinicians felt that the emotional and cognitive demands of ED work became intensely preoccupying. Some expressed distress due to constantly having to think about food, calories, and exercise, leading to heightened self-awareness outside of work and sometimes disrupting their own eating patterns or body image. Sometimes, this preoccupation crossed into disordered territory, whereas others developed a ‘resistance’ to diet culture.

*C5: When I did gain weight*,* I was very aware not to fall into diets. I’ve almost done it the opposite way. I’m worried that I would get into those unhelpful patterns… I’ve noticed that when I want to role model for the patients - I’m not maybe mindful of my own eating. So*,* then I gain weight*,* and that affects my own self-esteem*.

Clinicians also sometimes received direct comments about their bodies from patients, which could be perceived as intrusive or upsetting, particularly for those with heightened body image sensitivity. While these clinicians did not all identify as having lived experience of an ED, their accounts highlight how the emotional demands of ED work can intersect with personal vulnerabilities in ways that may resemble or even precipitate disordered patterns. The complexities of clinicians with lived experience are explored further in a separate publication.

#### Subtheme: perfectionism, self-criticism and confidence

Clinicians frequently displayed perfectionistic attitudes towards their work. Many were self-critical and doubtful of their own abilities, commonly experiencing impostor syndrome in their work.

*C17: Sometimes I think would having clearer treatment pathways would be helpful*,* but maybe it wouldn’t be. Maybe I’d just get stressed. I’d think I wasn’t doing that properly either.*

To reduce negative feelings, clinicians highlighted the importance of supervision, informal discussions, and self-reflection - strategies that helped to gain perspective and remember that no single clinician can be solely responsible for a patient’s recovery.

### Subtheme: emotional labour and coping

ED work was described as emotionally intense, both demanding and meaningful. Some clinicians referred to ED work as a “Marmite” specialty, requiring strong personal values and emotional resilience.

*C18: It takes a special person to decide [to start] working with EDs. Not everyone is cut out to do that … there is a high risk of burnout.*

Clinicians frequently encountered emotionally exhausting situations, observing the impact on their energy within and beyond the clinical setting. Many struggled to maintain a healthy work-life balance and noted how their emotional investment in patients’ recovery could risk overinvolvement. Boundaries were seen as essential to protect both clinician wellbeing and therapeutic integrity.

*C5: I get burned out I think quite routinely…when I’m at work I can lose sight of my own well-being because I’m just so focused on them.*

Some clinicians also reflected on a gradual desensitization to risk, viewing it as a self-protective response to the emotional toll of repeated exposure.

*C13: My team go and talk to people about their thoughts of suicide all day*,* every day - they can’t have the same emotional reaction that the physical health nurses [have] … they wouldn’t be able to do their job.*

Their emotional responses were not seen as weakness, but as an integral part of meaningful therapeutic work. The challenge lay in sustaining this emotional labour without neglecting one’s own needs. Coping strategies included mindfulness, grounding, and self-reflection - often adapted from the techniques clinicians used with patients. Social support was also highlighted as vital.

### Theme 4: team dynamics

#### Subtheme: synergy and cooperation

A strong MDT with a positive and supportive team culture was important for job satisfaction and a sense of safety at work. Joint working experiences fostered a sense of unity and mutual compassion among team members. Clinicians felt able to lean on their colleagues for support, e.g., when dealing with anger from patients’ families, or when working with a challenging case.

*C3: I think working with families … It can be quite tough if there’s a lot of anger coming at you - very articulate anger. I can feel quite intimidated. But that’s where for me*,* the team - the very strong team - can help support you with that and not feel alone with it.*

Clinicians also highlighted the importance of awareness of team dynamics and the systemic impact that every individual has on the team. Clinicians mentioned holding space for group-reflection and the need to be curious about their colleagues’ wellbeing, as the team dynamic and collective wellbeing equally shaped their individual wellbeing.

#### Subtheme: differences in opinion and communication conflicts

Clinicians’ diverse backgrounds and levels of experience brought various attitudes and perspectives to their work, sometimes leading to differences in opinion. Newer clinicians felt less able to voice their views around those with more established positions. Occasionally, clinicians expressed concerns about their colleagues’ attitudes. For instance, some clinicians perceived their colleagues to hold rigid attitudes toward autism and neurodivergence, which left them feeling uneasy. Other clinicians noted their differing views to colleagues who they perceived to hold more traditional or outdated attitudes.

*C12: I was recently diagnosed with autism. When I bring that up in services*,* there’s stiffness from a lot of the older staff. They say ‘Oh everyone has this nowadays’ … hearing the way they speak about patients who can quite closely resemble a background to myself - I was uncomfortable in those kinds of situations because it’s seen as [the patients] being disengaging or unable to comply with treatment…*.

Further challenges arose from pitfalls in team communication, often driven by management changes that led to confusion, inconsistent messaging, and increased anxiety. Tensions and conflict within teams often left clinicians feeling unsupported. This dynamic intersected with perceptions of supervision quality, leadership, and broader organisational support.

### Theme 5: supervision, management, and organisational support

#### Subtheme: constructive supervision interactions

Clinicians emphasised the importance of having protected time for supervision and its impact on their wellbeing. Having a safe and non-judgmental environment to express their needs gave clinicians a sense of security in their working environment.

*C15: Just in general - not necessarily just when something’s gone wrong but just knowing again that there’s protected time that I have to bring something where I can raise it with someone who’s got a different perspective or someone who’s got different knowledge …*.

Many supervisors also supported skill and career development from the outset, contributing to a sense of confidence amongst clinicians.

#### Subtheme: constraints on supervision and support

Clinicians recognized that increased service pressures were likely impacting supervision. Firstly, this was due to limited protected time for supervisory support. Secondly, clinicians hesitated to seek help, worrying about being too burdensome for already overstretched supervisors.

*C9: Our clinical lead is amazing. However*,* he is extremely overworked and very stressed*,* so whilst I know that I can go to him anytime I need the support… I don’t want to keep putting on him*.

The influence of available resources on supervision was evident. Giving more space for supervision can have positive impact on job satisfaction.

*C14: When I’ve worked in mental health before*,* I’ve got on with my supervisor… it’s just it didn’t click as well. It didn’t feel as fulfilling. It’s not because they didn’t want to be supportive*,* but I think it’s a lack of resources and time.*

Taken together, this points to the pivotal role of both individual supervision and systemic leadership for clinician wellbeing.

#### Subtheme: management hierarchies and communication gaps

While most clinician-supervisor relationships were positive, some felt workplace hierarchies reduced job satisfaction. They perceived a disconnect between management and frontline staff, with those in higher roles often unaware of day-to-day challenges.

*C6: [There is] quite a bit of frustration about a kind of separation between the clinicians and those who got more managerial roles… I am aware that that there is a feeling of ‘decisions get made about our team and we never get a say’ … there is a sense of being segregated …*.

Many clinicians expressed a desire for greater recognition from supervisors or management, believing it would boost job satisfaction, confidence, and a sense of reward.

*C8: just the acknowledgement of the amount of time it takes to conduct the work in terms of all aspects of delivering this therapy and care coordinating… how mentally taxing it can be.*

Others expressed disappointment that efforts to raise concerns often felt ineffectual, or that staff wellbeing initiatives were seen as hollow when not backed by action. At times, clinicians perceived managers’ interest in their wellbeing as disingenuous, particularly when their needs were repeatedly unmet. However, there was a desire from clinicians for greater understanding from their managers to bring out the best in the team.

### Theme 6: service-level concerns

#### Subtheme: Influence of service setting on clinician distress

The service setting sometimes influenced clinicians’ wellbeing, particularly in inpatient wards with patients with life-threatening illness unable or unwilling to comply with prescribed meal plans. In such cases, clinicians often had to resort to nasogastric (NG) feeding under restraint. Many described the emotional toll of this practice, recognizing its clinical necessity, yet feeling deeply distressed when administering or witnessing its use.

*C7: I think of one specific patient we had to NG feed — it was very traumatic for them … Their reaction was always screaming*,* shouting*,* swearing — saying things like ‘You’re killing me.’… I’d go into the room where we did the NG feeds to take my break — and I’d just feel really sick. I don’t want to say PTSD symptoms*,* but… just that emotional reaction to being in the environment where it happened.*

The conflicting emotions (empathy for the patient, distress from the NG feeding itself, and professional obligation) appeared to contribute to a lasting psychological impact on clinicians. Moving from working in an inpatient to an outpatient setting led to emotional relief for some; however, others felt that the emotional intensity remained in different forms. In outpatient care, the burden of ensuring patient safety in a community setting left some clinicians feeling equally drained.

#### Subtheme: workload pressures and the need for boundaries

Clinicians with higher job satisfaction often attributed this to manageable workloads. Smaller caseloads and lower referral volumes were seen as protective for wellbeing. However, most clinicians reported high caseloads and heavy responsibilities. Pressure to reduce waitlists created a sense of likely failure, especially for those with perfectionist tendencies, leading to dissatisfaction with their work. This strain, for some, was described as a reason to consider leaving their roles.

*C13: it’s very uncomfortable because you’re working well beyond in terms of what your actual remit is*,* in terms of your job probably*,* somewhat beyond your competency frankly. But it’s you or nobody*,* and without any other resource*,* well over your hours and dealing with incredibly risky*,* incredibly sick people*,* there comes a point where you have to say if nothing’s going to change - then to have work life balance - I might have to change my job. Which seems a shame*,* because then you leave behind a complete gap…*.

Long waitlists further contributed to distress. Knowing that patients deteriorated significantly while waiting for care without being unable to intervene led to feelings of helplessness. To cope with these challenges, clinicians created boundaries to protect their work-related and personal wellbeing. For some, these boundaries were self-initiated and refined over time. For others, support and modelling from managers were essential in learning to prioritise self-care.

*C8: I’ll make sure I take my lunch break*,* and I get out the building and just disengage from it. … I’ve learned over time*,* when I do have a spare moment*,* that’s OK*,* I don’t need to fill it with stuff.*

Some frustration was expressed about the lack of structural or institutional support for staff wellbeing, which left clinicians feeling the need to protect themselves from burnout.

*C8: We want to have more opportunities for staff well-being… engaging in sort of restorative activities as a team to take a breath before going back into the work…that’s kind of been rebuffed a lot from management*,* which is very frustrating. So*,* you essentially have to create your own boundaries- it doesn’t necessarily feel like the management will protect you from burnout and overwhelm.*

#### Subtheme: financial challenges and lack of resources

Financial constraints varied across services but consistently affected staffing, waitlists, and work-life balance. Clinicians were disappointed that limited resources sometimes prevented them from meeting patients’ needs.

*C6: We are in a Trust that’s having massive financial issues … We can’t buy any stationary at the moment … things like asking for training and staying professionally up to date is really challenging and makes us all feel a bit wary for the future. And we still need to expand… but if we can’t buy plastic wallets*,* we’re not recruiting anyone… There are criteria for what an adult ED service should be offering and we’re not meeting that… It’s not a skills problem; it’s just a human resources problem.*

Remuneration of work was also a concern, with clinicians questioning the financial sustainability of working in their current roles and the ethical dilemma of leaving the NHS for higher-paid work. Deficits in resources for ED services seemed related to a wider, shared issue across the NHS.

*C6: we’re not the only service that is understaffed and struggling … We know we’re not alone with that and I think that there is a degree of comfort in that.*

High staff turnover compounded recruitment and retention difficulties, further affecting clinicians’ workload and emotional strain. Early discouragement from ED work during training was identified as contributing to recruitment challenges.

*C19: When I was training… we were kind of discouraged from going anywhere near eating disorders. ‘Oh it’s too hard. You won’t like it. They don’t wanna get better. You’ll wear yourself out.’*

Some felt disheartened and alone in their work when seeing the lack of permanent staff on their teams. Clinicians acknowledged the potential effects that this might have on patients, for instance, not being able to build sufficient trusting relationship with their therapist or team. Clinicians highlighted the impact of staffing shortages on their work-life balance:

*C5: I didn’t really feel I could take any of my annual leave because then you feel like ‘Oh*,* what if someone gets discharged because of me?’ And then someone else takes their leave and that means there’s not enough staff …*.

Staff shortages related to concerns about service disparities across the NHS. Commissioning disparities meant some services lacked key roles, leaving clinicians with specialist training in generic posts. Even when training was available, staffing pressures often prevented attendance. Where services had resources to hire more staff, the benefits were tangible.

*C6: We initially tried to do [a course] one year and we were so short staffed*,* we couldn’t turn up and do it.*

#### Subtheme: training needs

Clinicians acknowledged that training needs vary by profession, role, and level of experience. While formal training in manualized, evidence-based ED therapies was seen as valuable, many felt it had limitations. Ongoing clinical development was also shaped by regular supervision, learning from experienced colleagues, and hands-on experience in day-to-day practice.

*C19: you theoretically can pick up any manual and figure it out as you go and that’s quite daunting because it was my first job as a qualified therapist. But I do think it’s the best way to sort of learn by doing and to realize that was just an essential kind of experiential part of the process.*

Some clinicians felt they lacked relevant skills, specifically mentioning meal planning and mealtime support as areas of concern. The inclusion of simulation training was flagged as potentially helpful.

*C17: I’d really like some more like meal planning because I maybe overthink it…I often use the safety net of a dietician to kind of go and support with that*,* I don’t think I’d feel that confident without it…*.

Another area for training expansion was Avoidant/Restrictive Food Intake Disorder (ARFID), as this is a relatively new diagnosis. Some services now offer assessment and treatment for ARFID, whilst others lack commissioning but wish to support this under-served population.

*C7: we need more money to open to people with ARFID. I think that that does come down to training because we don’t have the clinicians … we don’t have a pathway or really a treatment for ARFID…*.

Together, these service-level challenges were frequently rooted in broader systemic pressures.

### Theme 7: macro-level systemic concerns

#### Subtheme: systemic coordination difficulties

Clinicians identified challenges accessing referral and care pathways in other NHS mental health services for ED patients, particularly due to uncertainty about what those services offered.

*C2: we have too many working in silos in the NHS… I’ve not got any idea what that service over there does. I don’t know how I can support them*,* and they don’t know how they can support me.*

These siloed interactions were felt to create gaps in the system when trying to coordinate with one another. Clinicians also voiced concerns that services did not/were not able to consider mental and physical health together, treating the individual conditions rather than the patient as a whole – creating further treatment gaps.

*C13: I think that physical health services see these patients as psychiatric services. [Psychiatric] services see these patients as physical*,* and they fall into this gap in between. There’s a huge amount of collective ignorance about what they actually need.*

Where ED services lacked medical personnel, physical risk monitoring fell to primary care, requiring frustrating liaison. Furthermore, clinicians expressed frustration and concern about providing care to ED patients with substantial comorbidities that required separate intervention. These patients were felt to not be adequately served. Clinicians emphasized the need for increasing ED training for those outside of ED services to raise awareness about ED patients and ameliorate these coordination challenges.

*C6: I get quite upset and frustrated on the behalf of people that I’m working with who are sitting there in evident distress about things that are not under the remit of the eating disorder service and can’t access anything else - that doesn’t feel right. I suppose that is moral injury.*

#### Subtheme: regulation through policy and targets

Clinicians felt frustrated when unable to accept referrals below service criteria thresholds. Prioritising patients by severity was described as disheartening, especially when those deemed “not severe enough” faced delays. Clinicians acknowledged this as a necessary but morally difficult reality of NHS care.

*C6: whenever you prioritize an urgent case*,* you’re inevitably delaying the treatment of everyone else… It doesn’t feel like a choice. It’s just what we’ve got to do.*

Many advocated for broader access and earlier intervention, noting that entrenched symptoms become harder to treat over time. For those with early intervention pathways, the impact was tangible.

*C11: we’ve got an early intervention pathway*,* which has been brilliant. …you can get in and treat people within that kind of crucial window…I can see things have changed for the better.*

Clinicians felt pressured to deliver standardised care within limited sessions, often having to discharge patients prematurely. They attributed this to managerial priorities focused on reducing waitlists.

*C12: [a patient] couldn’t reach the targets that were set for the treatment*,* but she had a lot going on outside of that … She just took a little bit longer … it’s taking an individual basis of what people need. I think that’s sometimes lost in treatment pathways.*

Correspondingly, some clinicians voiced their concerns with how patients with longstanding illness are perceived, labelled, and treated.

*C2: Why are you referring to a patient in that way? Regardless of if somebody might have had liver disease for a long period of time. Does that mean that they get less? That didn’t sit right with me morally …somebody can recover from an eating disorder at any time.*

Clinicians experienced pressures from senior management, feeling the need to meet their targets, whilst having firm criteria for discharge – presumably stemming from resource constraints. Despite this demand for ‘more work for less,’ clinicians felt a lack of recognition for their diligence.

*C19: I wanna make sure this person is looked after even though we’re not the right service for them. But in doing so*,* I’m missing my supposed numerical target for people I’m supposed to see. The human side of me wants to support another human over meeting a target. But then the target has an impact.*

Disconnects between frontline staff and senior management contributed to a lack of clarity and support, with clinicians often left to compensate for top-down decisions made under resource strain.

In sum, clinicians highlighted how policy-driven constraints, referral criteria, and performance targets undermined the delivery of cohesive, person-centred care.

### Theme 8: broader societal challenges in ED care

Deep-rooted trends relating to diet culture and body image were seen as a broader barrier to ED recovery. Clinicians expressed the challenge of supporting a patient through recovery whilst societal pressures and thinness ideals constantly undermined their efforts.

The uptake of services by different demographic groups also raised questions about ED recognition by GPs or patients themselves, cultural or societal barriers to patients accessing care, or broader challenges in ED services and the NHS.

*C6: In our service we see more white middle-class females than our demographic would indicate should come through to our service with a mental health illness that could affect anyone. That is difficult for us because we*,* to some degree*,* are at the mercy of what’s happening in primary care.*

Clinician 4 referred to broader public awareness efforts to reduce stigma and misconceptions about eating disorders at a societal level. However, their reach can be limited when the message is mostly taken up by self-selecting audiences. This raises the challenge of how to disseminate accurate information more widely through population-level channels.

*C4: In reality*,* this takes a very long time to change society and societal conceptions… whether it was gender politics or stigma related stuff … Where do we need to target that message? We need to push it out further a bit. That’s gonna be GPs*,* wider society*,* schools*,* education…*.

Together, these broader societal and cultural dynamics impact not only patient access and recovery but also the clinician experience, contributing to systemic gaps in ED care.

### Theme 9: COVID-related changes

#### Subtheme: navigating service changes, discharges, and ED acuity

Clinicians noted that the onset of the COVID-19 lockdowns precipitated a surge in ED referrals to services, with the isolation, panic-buying, and increased media exposure likely being contributing factors. Likewise, these factors may have aggravated ED behaviours amongst existing patients or may have changed the ways in which EDs presented in certain individuals. For instance, some clinicians noticed an increase in ED presentations alongside personality disorders and self-harm.

*C16: the challenges have then become the acuity*,* the patients that we’re seeing now are just so unwell - it is a huge shift. And I would probably say along with that then - whether it’s the pandemic that contributed to it - is the risk and the self-harm. Now we’re seeing a lot more of that kind of EUPD presentation….*

Lockdowns required inpatient wards to discharge patients where possible, and some clinicians described moral injury in these discharge decisions, expressing their concern for patients’ welfare during this time.

*C5: A lot of people got discharged who were still unwell and they shouldn’t have been discharged - but they had to be because our staffing levels were so low that they did only keep the most ill patients on the ward. It didn’t feel right*,* even for those patients [still on the ward]*,* I guess they knew that they were the ones getting the care because they were the sickest. And I think in a way that almost fuelled some of their eating disorder behaviours.*

Services had to be restructured, such that clinicians experienced major changes in their roles. One managing clinician referred to the difficulties of having to make these changes, having to hold staff members’ fear and frustration.

*C2: I had to make difficult decisions about redirecting staff to other wards. And some staff were not happy about that and said that I was putting their lives at risk. Obviously*,* they were working on areas that did have outbreaks of COVID… So that was personally very difficult to deal with - sort of being told that I’m putting somebody’s life in danger.*

Other concerns regarded the challenges of delivering ED treatment virtually, especially with higher-risk patients. Monitoring weight, for instance, was challenging as some patients did not have access to scales or might have concealed any changes in weight with clothes or adjustment of their webcam.

#### Subtheme: residual challenges and lasting changes

Due to the largescale impact of Covid on worsening existing and precipitating new EDs, services struggled to manage their referrals and waitlists. Such an increase has had a residual impact on clinician wellbeing and burnout.

*C4: it was just about firefighting as opposed to doing quality - as a psychotherapist - what I would think of as quality work. It’s improving massively*,* but the dust is only just starting to settle. It’s taken a massive physical and psychological toll.*

However, there was optimism for the future of services, with some clinicians noticing the amelioration of such challenges.

*C7: There’s excitement now. We’re doing quite well at coming out of Covid and we’re in a point now where it’s still really high*,* but it’s coming down.*

Several of the changes established during the lockdowns have remained in place, with tangible benefits to clinicians and patients. A shift from solely in-person working to hybrid working has mostly been a positive change for many clinicians. The home working environment encouraged productivity and feelings of comfort for some individuals.

*C12: We work on hybrid kind of basis. I benefit from that myself as a neurodivergent person who sometimes doesn’t like being surrounded by a lot of the noise that comes from being in those situations.*

Reduced commuting and more accessible virtual appointments also improved work-life balance for clinicians and patients alike. However, remote work reduced some informal interactions, termed as the informal ‘water cooler’ exchanges that build rapport and collaboration, prompting clinicians to encourage more regular in-person time.

*C3: Every interaction is an intentional one because of the meeting*,* rather than the more liminal spaces in between*,* where you can sort of debrief or humanize each other a bit…They restore you to a human being in a way.*

While the COVID-19 pandemic introduced significant disruption to ED care, it also catalysed lasting changes—some of which have improved work-life balance and accessibility, while others continue to challenge the capacity, wellbeing, and cohesion of ED services.

## Discussion

Nine themes evolved from the data: [1] clinician motivation for working in ED services [2], complexities of ED management [3], clinician personality and emotional disposition [4], team dynamics [5], supervision, management, and organizational support [6], service-level concerns [7], macro-level systemic concerns [8], broader societal challenges in ED care, and [9] COVID-related challenges. These themes were then organized into five supra-themes, influenced by Bronfenbrenner’s (1979) Ecological Systems Theory, forming a holistic ecological systems framework to contextualize ED clinicians’ current experiences. Key concerns included the chronic nature and risk of EDs, growing service demands amid limited resources, and service-level and commissioner-imposed targets.

### Intrinsic influences

ED clinicians were highly committed to their roles, driven by sustained interest in the field and reinforced by personal fulfilment. Several studies similarly document these strong value-driven attitudes among ED clinicians, as well as positive perceptions of their work and patients [18, 27, 28]. Despite the emotional demands and challenges of ED care, clinicians maintained belief in the effectiveness and value of their interventions [27, 29]. This intrinsic drive may offer some protection against burnout [11].

Clinicians’ experiences were also shaped by individual emotional responses, personal standards, and perfectionism. Their capacity to manage complex ED care was influenced by personal susceptibility to burnout and the coping strategies they used to mitigate emotional exhaustion, influencing their susceptibility to burnout and the coping strategies employed. These dynamics reflect countertransference, where clinicians’ perfectionistic traits mirrored those of their patients [18]. Together, these intrinsic motivations and personal dispositions form the individual-level foundation upon which clinicians navigate the broader challenges of ED care.

### Intrapersonal factors

Challenges included patient attachment to the ED, variability in motivation for recovery, and high-risk or acuity, as noted in previous studies [11, 30]. These challenges were highlighted in Hyam et al. [31], where the increasing acuity and complexity of post-Pandemic ED presentations had significant implications for service access in the UK. Some clinicians received direct comments about their bodies which could be perceived as intrusive or upsetting, potentially affecting clinicians’ own relationships with their body and/or food. One study similarly reported that most ED clinicians received commentary about their appearance from patients, and subsequently reported increased vigilance or self-criticism about their eating behaviours and appearance afterwards [27]. Moving beyond individual clinician characteristics, the relational dynamics of the team environment emerged as equally significant in shaping clinician wellbeing.

### Departmental factors

Team dynamics shaped clinicians’ job satisfaction. Supportive peer networks and constructive supervision emerged as key protective factors. However, differences in clinical opinions, communication difficulties, and limitations in supervisory support occasionally detracted from clinicians’ job satisfaction. The importance of high-quality supervision that explicitly addresses burnout and countertransference reactions, and that reinforces appropriate professional boundaries in responsibility for patient outcomes has been previously highlighted [27, 28]. Whilst supportive team environments offered some buffer against occupational stress, these were frequently undermined by wider organisational and systemic pressures.

### Organisational and systemic challenges

Pressures on services remain elevated since the pandemic [32], affecting clinicians’ job satisfaction and emotional exhaustion. Financial challenges within NHS adult ED services have been well documented [33], with this study further demonstrating the continual impact of this on clinicians. Implications for the workforce are significant, with persistently high workloads increasing the risk of burnout, sickness absence, and potentially staff turnover, further reducing service capacity and creating a cycle of demand exceeding resources.

Moreover, the influence of the service setting was highlighted, with clinicians expressing the challenges accompanying both inpatient and outpatient settings, as well as differences between public and private healthcare systems. Warren et al. [30] found lower levels of burnout amongst clinicians in private practice. The present study similarly reports that some clinicians view private practice as more favourable in terms of renumeration and resource availability. Additionally, though not said in the interviews, it must be noted that providers in the private sector typically have greater discretion over which patients they accept under their care. This contrasts with public sector providers (e.g., NHS), which are legally obligated to provide care to all individuals in their area. This contextual distinction may influence clinician experiences and wellbeing, although was not a direct focus of this study.

Another issue concerns intra- and inter-service coordination of care, and how this affects patients and clinicians. Similarly to Warren et al. [34], clinicians expressed frustration about coordinating with the primary care sector, noting that the unclear delineation of roles and responsibilities of each clinical team often placed strain on services. This lack of clarity left clinicians feeling solely accountable for aspects of care that should be/could be shared, such as physical health care or early intervention efforts. Poor communication and connection across services complicated care continuity, leading to fragmented treatment pathways. These difficulties create gaps in care that increase risk for patient deterioration during transitions between services [35].

Systemic siloing exacerbated clinician frustration and distress. EDs are unique in that the overlap between physical and mental health is central to their presentation, although this may have inadvertently reinforced excessive siloing in clinical care [36]. EDs are marginalised within mental health services, where non-ED clinicians may feel ill-equipped to manage these disorders, ultimately placing the patient in a state of service ‘limbo.’ For example, when patients with EDs and comorbidities require care beyond their ED, general mental health professionals may decline involvement, stating they ‘do not treat EDs’. The recurring theme of ‘juggling risky patients’ reflects a pervasive stigmatisation and fear surrounding EDs, both within ED services and across health services more widely, contributing to risk aversion and the passing of clinical responsibility.

Clinicians felt constrained by rigid service criteria, which sometimes resulted in premature discharges or patients being denied treatment if they did not meet diagnostic thresholds. Whilst clinicians voiced a desire for a less target-driven culture, they also indicated that clearer service policies could help reduce role-related uncertainty [10]. These structural constraints had tangible ethical consequences, with many clinicians describing experiences consistent with moral injury.

Several clinicians alluded to feelings of moral injury in their work – for example, having to deliver lifesaving but coercive and distressing feeding practices. Notably, the dilemma of how to best allocate resources in the context of an under-resourced NHS was evident. These references to moral distress align with research in other healthcare settings. In such contexts, clinicians are unable to act in accordance with their professional or personal values due to service constraints [37].

Three ethical principles are typically applied to guide such healthcare decisions [38]. The first is egalitarianism, in which everyone has equal access to healthcare across the population regardless of disadvantage. The second is utilitarianism, which attempts to direct care to those who would benefit the most, such as those in early-stage illness who would be more likely to respond to intervention. Third is prioritarianism, which focuses on allocating resources to the most severely ill or unwell individuals [39]. Clinicians tended to agree that the most medically severe patients need to be seen urgently, whilst recognizing the importance of early intervention irrespective of severity. However, there was concern that physical metrics, such as BMI, are overemphasized in ED treatment – suggesting that symptom severity, broader risks, distress, or impact on functioning should be prioritised instead. Brief programme-led and task-sharing interventions, such as guided self-help [40], are avenues to reduce service pressures, though more needs to be done in terms of ED resourcing overall. In summary, this highlights the tension between competing ethical frameworks in ED resource allocation and the need for a more holistic, multidimensional approach to triaging care.

Concerns about clinician confidence and knowledge in specific areas of ED care underscored the importance of comprehensive team training [12]. Clinicians identified a need for greater education on meal planning and support, medical risk assessment and management, and on assessment and treatment of ARFID. Evidence suggests that whole-team training interventions can effectively improve both confidence and competence in managing ED cases [24]. A further consideration is the extent to which these findings reflect challenges that are inherent to EDs themselves, versus those that are primarily driven by systemic factors such as limited resources. Some of the difficulties described are unique to ED services because of the unique clinical complexities of EDs. For example, the acutely life-threatening nature of many severe ED presentations, the ambivalence of some patients towards recovery, the use of lifesaving but coercive feeding practices, and the prolonged course of illness may place additional demands on clinicians beyond those experienced in other mental health specialties. These findings must be considered within the context of the NHS, where resourcing for adult ED services in England remains constrained and has been further challenged since COVID [7]. However, it is likely that many issues raised here also affect publicly funded healthcare systems internationally, with increased demands on services since COVID being ubiquitous and well-documented. Beyond the immediate service context, clinicians’ experiences were also situated within broader societal and cultural landscapes that shape both the prevalence and treatment of EDs.

#### Societal contexts

Socio-cultural factors, such as attitudes towards body image and eating behaviours which likely shape both clinician perceptions of themselves, their roles and the treatment of EDs more broadly, are widespread across all WEIRD (Western Educated Industrialised Rich Democratic countries) - though are not confined to these settings. A growing body of literature highlights diverse and significant drivers of EDs in non-Western populations, including culturally specific body ideals, familial expectations, religious practices, and media exposure at varying stages of acculturation [41]. These factors likely shape both clinician perceptions of their roles and the treatment of EDs more broadly. However, similarities between our findings and those reported in international studies [29] suggest that many aspects of ED clinician burden and moral distress may be universal in this field. Taken together, these findings illustrate how clinician burden in ED services operates across multiple, interconnected levels.

Our analysis highlights how themes interconnect to form a nuanced narrative of clinicians’ experiences. For instance, *Fulfilment and Personal Growth* was sometimes overshadowed by the emotional strain described in *Coping with Patients’ Varying Treatment Motivation*. Similarly, *Chronicity and Stagnation* and *Spinning Many Plates* converge around clinicians’ efforts to contain clinical and systemic burdens, reflecting the cumulative demands of managing complexity, risk, and resource constraints. These interrelationships align with literature on emotional labour and burnout in mental health settings [1, 9, 13].

#### Implications

Sustaining flourishing ED teams requires adequate resources, emotional support, and systemic investment. When clinicians feel valued, supported, and resourced, they are more likely to remain in their roles and contribute to a stable, skilled workforce. In the absence of these conditions, prolonged emotional exhaustion can erode team morale, deter clinicians from remaining in specialist ED roles, and intensify existing staffing challenges across the field. Systemic interventions are needed to sustain the workforce to improve both clinician wellbeing and patient outcomes [13]. This might include providing protected time for supervision, and improved resourcing and allocation. Beyond consistent supervision and resourcing, effective strategies may include multidisciplinary collaboration to distribute responsibility across teams, peer-support programmes that provide spaces for reflection and mutual validation, and flexible workload management approaches [40, 42]. Such strategies might reduce burnout, enhance team cohesion, and support care delivery. In sum, teams require consistent supervision and collaborative structures, services must be resourced to meet rising demand, and health systems must invest in sustainable workforce models. Future research should longitudinally investigate the trajectories of burnout, job satisfaction, and job retention amongst ED clinicians.

#### Limitations

The national training course from which participants were recruited is NHS funded (i.e. free for clinicians across England having the opportunity and being expected to send their staff) is likely to be representative of ED clinicians across the UK. However, only a small proportion of these chose to participate. Most participants identified as Female and White/White British, keeping with the demographics of the wider pool of training participants. This limits the ability to compare whether gender or ethnicity might influence clinicians’ work-related experiences. It is also possible that those with a high degree of burnout may not have responded to the invitation to interview, either due to emotional load or high workplace demands. Alternatively, it could be that clinicians disproportionately affected by their work were more inclined to take part in this study. However, as the range of experiences and views was varied, this suggests that we captured clinician voices across the spectrum from high to low well-being and with varying degrees of burnout. Finally, as thematic analysis is interpretive, the construction of themes will have been shaped to some degree by the researchers’ own perspectives and theoretical assumptions, which represents an inherent hermeneutic limitation of the method.

## Conclusion

This study illustrates the interplay of factors influencing ED clinicians’ experiences in the UK. Through this lens, the findings reveal how internal motivation, emotional resilience, and professional values interact with external pressures such as limited resources and structural constraints. These areas can be targeted to improve clinician job satisfaction and reduce burnout risk, with the goal to provide optimal patient care.

**Table 1:** Participant characteristics

ID	Gender	Role	Service	Experience in Role	Experience in Service	LE	Interview length (mins)
1	F	Nurse	Outpatient Service	1–3 years	7 + years		62
2	F	Manager	ED Day Unit	4–6 years	7 + years		45
3	F	Consultant Clinical Psychologist	Adult Community ED Service	1–3 years		Symptoms of disordered eating	29
4	M	Specialist ED Mental Health Practitioner	Adult Community ED Service	7 + years		Yes	96
5	F	Mental Health Worker	ED Day Patient Service	4–6 years			37
6	F	Clinical Psychologist	Adult ED Service	1–3 years		Yes	44
7	M	Clinical specialist	Adult Community ED Service	< 1 year	7 + years		24
8	F	Specialist MHP	Adult Community ED Service	1–3 years		Yes, as carer.	33
9	F	Community Support Worker		1–3 years	7 + years	Yes	38
10	F	Counselling Psychologist	Adult ED Service	1–3 years			31
11	F	Nurse	ED Community Team	1–3 years		Yes	45
12	F	Assistant Psychologist	Adult ED Service	< 1 year	1–3 years	Yes	30
13	F	Consultant Liaison Psychiatrist	Adult ED Service	1–3 years			51
14	F	Peer Support Worker	0–25 ED Service	1–3 years		Yes	31
15	F	Assistant Psychologist		1–3 years		Symptoms of disordered eating.	31
16	F	Dietitian	Steps Community Adult ED Service	1–3 years	4–6 years		30
17	F	Nurse	Adult ED Service	1–3 years			34
18	F	Family Therapist	All-age ED Service	1–3 years	7 + years		52
19	F	Cognitive Behavioural Psychotherapist	Outpatient ED Service	< 1 year	4–6 years		33

## Electronic Supplementary Material

Below is the link to the electronic supplementary material.

Supplementary Material 1.

## Data Availability

The data that support the findings of this study are available from the corresponding author, K.N., upon request.

## Appendix A

### Interview schedule

**Table:**

Professional Experience	Professional Needs, Job Satisfaction
What is your professional role and how long have you been in this position?	What works well and not so well about the support you receive from your team and your managers? What is the support like? What can be done to improve the support you receive?
What enticed you to start working with eating disorder patients?	Have your support needs changed since the start of the pandemic? How?
What do you feel are some of the challenges and rewards of working in eating disorder services?	Is there anything else that affects your job satisfaction, positively or negatively (probe for: workspace and homeworking)?
Compared to before the pandemic, have there been any changes in your service and in your role? What impact has this had on your quality of life (health and wellbeing), both at work and outside of work (probe for: waitlists, referrals, rationing to certain patients, working online, managing risk, number of vacant posts, different/flexible ways of working)? How has this affected your relationships with work colleagues and management? How has this affected your work with patients and families?	If we could wave a magic wand and change one thing to improve your wellbeing and job satisfaction, what would it be? (what is the most important thing that we have discussed? )
How do you view the future of the service, the wider NHS and your role within it (e.g., changes in roles, reduced working hours)?	If you have encountered any situations in which you experienced moral injury, could you please describe it? How have they impacted your professional and personal life?
**Training Experience**	**Personal and Lived Experience**
What was your overall experience with the ML training? What did you most enjoy? What did you least enjoy? (probe for: content, mode of delivery, PPI involvement)	Do you have a lived experience with an eating disorder? If yes, are you comfortable with answering some questions about your experience?
What was your biggest takeaway, either positive or negative? Were there any unexpected learnings?	Could you please describe your experience; kind of eating disorder, treatment process, length of recovery, experience with the service, age at which started/diagnosed
Was there anything that influenced your practice? If so, how has your practice changed as a result?	What are your views about being open about your experience with colleagues or patients (probe for: how open, in which contexts, what details/how specific, what have patients’ responses been)?
Was there anything that you would have liked to see more or less of?	How has your experience affected your professional life?
What can be done to improve the training (probe for: gaps in content or delivery, PPI involvement)?	Do you ever feel affected/ “triggered” by patients? If so, in what ways? How do you deal with this?

## Appendix B

### COREQ checklist

**Table:**

Number	Item	Description
1	Interviewer/facilitator	KN conducted all interviews (P. 9)
2	Credentials	KN – MSc. CLL – MSc. JT – OBE, PhD, FRCP, FRCPsych US – OBE, PhD, MD, FRCPsych
3	Occupation	KN was a student researcher, and CLL was a research volunteer at the time of the study.
4	Gender	All authors identified as female.
5	Experience and training	KN had previous experience with conducting qualitative interviews and has studied qualitative methods.
6	Relationship established	No, participants were unknown to the researchers.
7	Participant knowledge of the interviewer	KN briefly introduced herself and her background. The information sheet outlined the study’s aims and publication plans. Lived experience was not disclosed during interviews (P. 9).
8	Interviewer characteristics	Reflexivity statement in methods section (P. 10).
9	Methodological orientation and Theory	Reflexive thematic analytic approach (P. 10).
10	Sampling	Convenience sampling (P.8).
11	Method of approach	Participants contacted via email (P.8)
12	Sample size	*N* = 19
13	Non-participation	One participant did not attend after providing written consent, no reason provided.
14	Setting of data collection	Interviews conducted virtually via Microsoft Teams (P. 9)
15	Presence of non-participants	No non-participants were present during interviews.
16	Description of sample	Participant characteristics reported in Table 1 (P. 8)
17	Interview guide	Interview guide provided in Appendix A (P.38)
18	Repeat interviews	No repeat interviews conducted.
19	Audio/visual recording	All interviews were audio/video recorded (P. 9).
20	Field notes	No field notes taken during interviews.
21	Duration	Interviews ranged from 24 to 96 min (M = 40; Mdn = 34; P. 9).
22	Data saturation	Data saturation was discussed and considered during analysis. The study aimed to recruit 20 participants.
23	Transcripts returned	Transcripts were not returned to participants for comment or correction.
24	Number of data coders	One primary coder (KN); themes were collaboratively reviewed and refined with co-authors.
25	Description of coding tree	Presented in Supplement 1.
26	Derivation of themes	Themes were inductively derived from the data.
27	Software	NVivo, Microsoft Word, and Excel were used to manage and analyse data (P. 9).
28	Participant checking	Methodology and results were shared with participants (P. 9).
29	Quotations presented	Participant quotations were used to illustrate themes.
30	Data and findings consistent	Findings were supported by data and consistent across the dataset.
31	Clarity of major themes	Major themes were clearly presented and discussed.
32	Clarity of minor themes	Subthemes were also described.

## Abbreviations

ARFID: Avoidant/restrictive food intake disorder
BMI: Body mass index
BRC: Biomedical research centre
ED: Eating disorder
MDT: Multidisciplinary team
NG: Nasogastric
NHS: National Health Service
WEIRD: Western, Educated, Industrialised, Rich and Democratic
