# Supplementary material for: “I can lose sight of my own well-being because I’m just so focused on them”: a qualitative investigation of eating disorder clinicians’ experiences in England
Source: J Eat Disord. 2026 May 2;14:166. doi: 10.1186/s40337-026-01615-9 (PMC13371212; doi:10.1186/s40337-026-01615-9)
Supplement: Supplementary file 1 — Supplementary Material 1. [file 40337_2026_1615_MOESM1_ESM.docx]

**Supplement 1. Coding tree.**

| Supra-Theme | *Theme* | *Subtheme* | *Example Codes* | *Example Quotes* |
| --- | --- | --- | --- | --- |
| **Intrinsic** | **Clinician motivation for working in ED services** | **Interest in a challenging but stimulating field** | **Relating to patients’ struggles**  **Mental-physical health overlap** | ***C15: I always had a theoretical interest in eating disorders when I was at university and I think it's a quite relatable topic, especially as a young woman.*** |
|  |  | **Feelings of fulfilment and personal growth** | **Building rapport**  **Seeing change and recovery** | ***C8: It's really rewarding when you see people get their spark back, because they come to you and that spark is very dimmed, they don't feel like themselves anymore. There's a real divorce between the self and the body - when that starts to become reconnected again, you can see that person coming back to life almost.*** |
| **Intrapersonal** | **Complexities of ED Management** | **Chronicity, stagnation, and treatment ambivalence – from frustration to hope** | **Longevity of the disorder**  **Ego-syntonic nature of illness**  **Feeling coercive** | ***C19: One of the challenges, and there's always a healthy dose of realism about that, is that [improvement] doesn't necessarily mean it's forever, doesn't mean it's cured. It might just be momentary.***  ***C6: It can be really challenging when people are in that very ego-syntonic position … they're like ‘we've done all this motivational work, but I'd still rather have it than not’…. Where do we go with that? We're not here to force someone to do something, but equally …we know how eating disorders affect how people perceive things and how just flipping awful and sticky they are … the eating disorder seems stronger than we are.*** |
|  |  | **Spinning many plates: managing patient risk and complexity** | **Managing medical risk**  **Juggling risky patients** | ***C9: There's always the looming, especially with the patients with lower weights, ‘what if they end up in coroner’s court…?’ From experience, the people who have been to coroner’s court have never really come back the same and they lost all their confidence.***  ***C17: there's so much to think about with eating disorders, you're trying to spin so many plates… trying to build therapeutic relationship with the young person, the family support system and how that looks like… their functioning in school …*** |
|  | **Clinician Personality and Emotional Disposition** | **Personal vulnerabilities to burnout and ED symptoms** | **Constantly thinking about food**  **Mirroring patients** | ***C5: having to think about [calories] all the time - constantly comparing what they're eating and also trying to like role model when I'm with them, so maybe having high sugar, high fat foods… I think in terms of my engagement, maybe with food, it has affected it … maybe I wouldn't have kind of thought about that before.***  ***C3: I've had it more in the CMHT when … a patient was doing a lot of, I don't know, looking at your body, that would probably be quite triggering for me, and I'd have to proactively push that away. Or if somebody was saying you're fat or something. Even if I knew that was just, they're in distress, that sort of thing would probably linger for me - even if it shouldn't.***  ***C4: I'd been keeping - just to learn - how to keep a weight diary and excel spreadsheets to weight charts for patients and stuff... I started to do that. And I was preoccupied by exercise. Those ED features started to surface with a preoccupation with weight and shape.*** |
|  |  | **Perfectionism, self-criticism, and confidence** | **Impostor syndrome**  **Reflecting on self-criticism** | ***C17: I think, when there's limited progress or when there's deterioration… I feel like I'm not doing a good enough job and doing something wrong.***  ***C14: you’re just kind of holding on to those moments…It's part of the whole wider journey of treatment and recovery - you're just one link in the chain, there's plenty of other links in the chain as well. You know you don't have to change their life overnight or anything - it's just being part of that.*** |
|  |  | **Emotional labour and coping** | **Taking home one’s work**  **Emotional overinvolvement** | ***C9: In the evenings, I'm really low in energy and motivation and I'm really, really tired because the days are– they’re only 8-hour days - but they feel really long and they're really mentally draining.***  ***C1: you're always wanting to do that little bit more for someone because you care … but you can fall into overstepping the mark … You create that dependence on a service… with both inpatients and outpatients - they have that reliance on the team... It’s sort of that mix between supporting someone and doing all you can but then either not colluding with the illness, then also not overstepping…***  ***C15: it's finding ways to sort of reassure yourself about what it is you're worried about. And I guess just applying psychological thinking the way you would with the patient and to try and minimize that.*** |
| **Departmental** | **Team Dynamics** | **Synergy and cooperation** | **Importance of team support**  **Systemic impact of team changes** | ***C9: the most important thing in the team is how close knit we all are… we've all got shared experiences and nobody else gets it... nobody else understands what we have to go through on a day-to-day basis… that support network between each other is just absolutely invaluable.***  ***C10: Next door there's a psychiatrist. The other door is a dietitian. And then you can be in close contact with all the other services in the community and that creates a sense of safety for me.***  ***C3: We have reflective practice … and a team peer supervision … we try I suppose to be mindful of those stresses or forces working on the team.*** |
|  |  | **Differences in opinion and communication conflicts** | **Differing opinions**  **Outdated attitudes** | ***C5: I guess when it comes to having to, as a band four, sit with decisions that I don't agree with that were made higher up. It's kind of trying to balance that ‘OK, this is the kind of decision of people that are more qualified than me.’***  ***C11: There are maybe a smaller number of people on the team who have slightly older fashioned attitudes… their views, I think, are outdated.***  ***C9: Communication isn't always the best … it can just cause a bit of confusion. We've had a couple of hiccups since some of the management changes and that's caused quite a lot of anxiety in the team - because we're kind of being told one thing and then someone else comes in and says something different.*** |
|  | **Supervision, Management, and Organisational Support** | **Constructive supervision interactions** | **Protected supervision spaces**  **Supportive supervisors** | ***C19: My service lead as well has put me forward for lots of additional training and allow me to incorporate my own areas of interest into this work … It's a really good place to grow and develop great colleagues.*** |
|  |  | **Constraints on supervision and support** | **Influence of resources on supervision**  **Not wanting to bother one’s superiors** | ***C15:* *I don't think there's always the best sort of opportunities for support within the team … I just think it's a time constraint thing above anything. I don't think it's necessarily advocated for as much as maybe patient care is, even though I feel like it's just as important...*** |
|  |  | **Management Hierarchies and Communication Gaps** | **Divide between frontline and managerial clinicians**  **Ineffectual communication with management** | ***C8: just the acknowledgement of the amount of time it takes to conduct the work in terms of all aspects of delivering this therapy and care coordinating… how mentally taxing it can be.***  ***C6: if you do wanna understand why people are feeling frustrated, just come and talk and have some conversations and people will value that. And maybe think about some of the things that are being done in terms of team development.***  ***C2: There are still hierarchies out there in the NHS … people sort of imply that everybody's equal and everybody's opinion’s valid - but sometimes you don't always feel like that, really. Because if I did say ‘I would like to do this’ I’m sure the bureaucracy would come in and it would be probably ‘no we won't do that’.*** |
| **Systemic** | **Service-Level Concerns** | **Influence of service setting on clinician distress** | **NG feed distress**  **Ensuring outpatient emotional containment** | ***C5: moving to outpatients, it's a lot of emotional containment because then [the patients] are going home at the end of the day. So obviously they're really distressed, you have to kind of take the time to make sure that they're OK before they go home. And I think sometimes it has just been like a lot coming back [home] and just feeling emotionally kind of drained. Like I don't have any more words to give almost to anyone.*** |
|  |  | **Workload pressures and the need for boundaries** | **Long waitlists**  **Difficulty setting boundaries for own wellbeing**  **Supervisors endorsing boundary setting** | ***C7: it's so difficult if you do an assessment with someone and you can see how desperate they are for an intervention and then having to add them to a waiting list, going back to that person explaining to them it's going to be a wait.***  ***C5: the first year I was always staying late, never taking my lunch break and just so, so focused on what the patients are doing and what the patients want, it just wasn't sustainable. I had quite a good manager who pointed that out … because she was so boundaried, it helped me to establish those boundaries for myself.*** |
|  |  | **Financial challenges and lack of resources** | **Making the most out of available resources**  **Renumeration of work**  **Some specialist roles not commissioned** | ***C7: Just having like more resources. I feel like that's always the main thing is what it comes down to, isn't it? … to have more manageable caseloads and to offer interventions…***  ***C4: Even the new people now, most of them are talking about leaving as soon as they can… That leaves me thinking ‘I'm gonna be the last one standing’ and I'm just exhausted and broken as a result.***  ***C9: the morale of the team over the past year has gotten so much better, and we got a load of new staff... we had over a years’ waiting time. And now it's more like 6 to 8 months, and that is because of the increase in staff.***  ***C6: We're in a team where people may have quite generic work roles but come from a specialist background … they can't exercise what they're really trained to do.***  ***C19: I hear lots of people, they're making so much money in private practice because it's very, very much in demand… But I really believe in the values of the NHS. So, I do wanna work with them. It's a very imperfect system, but it's still an incredible system that I can't believe it exists…***  ***C14: Our service is pretty good, but it is so focused on physical recovery, and I think that's because of time and resources more than anything…Once the person’s kind of got to a point where they're ok, but they're still experiencing a lot of psychological thoughts and stuff, they will still be discharged.***  ***C16: the inpatient unit has got about 5 to 7 original full-time substantive posts and then the rest are bank and agency, which is just so sad for the patients because they cannot hold the boundaries that they need. The lack of knowledge that the staff have just because they didn't work on inpatient units.*** |
|  |  | **Training needs** | **Lack of mealtime/dietetic training**  **More on ARFID** | ***C8: I guess the thing is having the supervision to implement it all the time and space to implement it. So, I've done the family therapy for anorexia training, but I wouldn't feel confident implementing that unless I'd shadowed somebody.***  ***C5: even mealtime training, we don't really have that. we've all just learned from each other… it would maybe be nice or helpful to have someone who's experienced to do a mock or even like role playing…*** |
|  | **Macro-Level Systemic Concerns** | **Systemic coordination difficulties** | **Working in silos**  **Patients falling in between the gaps** | ***C10: It's really difficult to handle the number of services that are in the community… maybe that’s because I come from another country and I'm not familiar with that…all the different IP BBTS CMHT MLHT... OK, so I'm discharging a patient. Where do I sign post? They still need therapy - where to send them? I think that we need a better understanding of all the different services that are out in the Community so that we can meet our patients’ needs after they recover.***  ***C8: one of the biggest challenges is managing the physical health, the mental health and the time it takes to liaise with doctors. We don't have phlebotomy in-house, so we have to liaise with GPs, and it takes so much time…We can't do the blood tests… but then our service has a responsibility to interpret them, and sometimes that can fall onto you as a practitioner…I don't feel comfortable doing that… there's a sense of ‘who is actually holding [the responsibility]?’***  ***C19: There's the lack of safeguards and then falling between the gaps in services so many times, I see people who have an eating disorder alongside many other critical sorts of life or mental health disorders.***  ***C13: You can’t care for physical health without considering mental health. Working with ED patients has improved the whole ward’s practice…across all patients...*** |
|  |  | **Regulation through policy and targets** | **Patients classed by severity**  **Financial targets for the service** | ***C2: our service has to look at people in severity categories - do we need to see you quickly or can you wait? - and that's always really difficult. If I've gone to the GP to, you know, to say ‘I've got a problem… I would like to be seen in a timely manner’. but understanding that whatever I'm being seen for, there'll be a waiting list and there might be people in that area lot sicker than me. When it comes down to it, it is about that one person, and that person wants to be seen.***  ***C8: our service is commissioned for bulimia and anorexia only …those patients that [have] disordered eating, emetophobia, currently ARFID … There's nowhere for those people to go, really*.**  ***C9: Progress was very minimal… but she was making very small changes, and I was basically put in a position where it was like, ‘no, you've worked with this patient for a long time now… She needs discharge’. I really didn't want to ... I just felt awful. It was one of those things where I thought, ‘what is the point in doing this?’ I remember coming away feeling like I'd let her down. Talking about it now, I feel that I've got a sadness in my chest, but it's just obviously how it is.***  ***C8: more recognition from management about the amount of time and work it takes with the patients is - and I don't mean that in terms of [clinical] - I mean that from a service management level - upwards service management, prescribed services - so manager, commissioner type level. It almost feels like the head is disconnected from the body.***  ***C13: They're just saying that they don't have any money, and they can't agree on a model and in the meantime, those of us who are on the frontline are having to do the work...*** |
| **Societal** | **Broader Societal Challenges in ED Care** |  | **Pressures on appearance**  **Patient access to care** | ***C15: societal pressures that are just impossible to mitigate. It feels really hard to argue with that as a clinician because it's just perpetrated everywhere. How am I gonna respond to that with a patient? I always feel stumped because it's like ‘my words realistically are not gonna measure up against you going home and sitting on your phone for hours and getting hundreds of different messages from hundreds of different places that tell you the complete opposite of what I've just said.’*** |
|  | **Covid-Related Challenges** | **Navigating service discharges and ED acuity** | **Discharging risky patients**  **Shifts in presentations** | ***C16: the challenges have then become the acuity, the patients that we're seeing now are just so unwell - it is a huge shift. I would probably say along with that then - whether it's the pandemic that contributed to it - is the risk and the self-harm. Now we're seeing a lot more of that kind of EUPD***[***^12]^***](https://applewebdata://EC65B879-681D-4603-9C1C-DB8CF46A1A0D#_ftn2)***presentation.***  ***C2: there's a lot of patients that you do need to see face to face, that can be difficult when people are hiding behind cameras.*** |
|  |  | **Residual challenges and lasting changes** | **Lasting impact on waitlists**  **Flexibility of hybrid working**  **Challenges of hybrid working** | ***C10: it was the first time that this service had to deal with the waiting list for assessment. Everybody was panicking. This huge pressure was created on us to work through the waiting list. We managed to do it, but at what cost? The moral injury and the burnout …***  ***C16: simple things like putting a wash on or finishing at four and you’re at home and you can go straight out and do a food shop or whatever you've got to do. I would say my work life balance is probably one of the best it's been.*** |

[**^[1]^**](https://applewebdata://EC65B879-681D-4603-9C1C-DB8CF46A1A0D#_ftnref2) **Emotionally Unstable Personality Disorder**
